# Oxidative Stress and Psychiatric Symptoms in Wilson’s Disease

**DOI:** 10.3390/ijms26146774

**Published:** 2025-07-15

**Authors:** Grażyna Gromadzka, Agata Karpińska, Tomasz Krzysztof Szafrański, Tomasz Litwin

**Affiliations:** 1 Department of Biomedical Sciences, Faculty of Medicine, Collegium Medicum, Cardinal Stefan Wyszynski University, Woycickiego Street 1/3, 01-938 Warsaw, Poland; 2 Department of Experimental and Clinical Pharmacology, Medical University of Warsaw, Banacha 1b Str., 02-097 Warsaw, Poland; 3 Department of Psychiatry, Faculty of Medicine, Lazarski University, Anna Gostyńska Wolski Hospital, Marcina Kasprzaka 17 Street, 01-211 Warsaw, Poland; 4 Department of Neurology, Stroke Unit and Rehabilitation Subunit, Anna Gostynska Wolski Hospital, Marcina Kasprzaka 17 Street, 01-211 Warsaw, Poland; tomlit@medprakt.pl; 5 Second Department of Neurology, Institute of Psychiatry and Neurology, Sobieskiego Street 9, 02-957 Warsaw, Poland

**Keywords:** Wilson’s disease, copper, oxidative stress, manganese superoxide dismutase, glutathione peroxidase, catalase, genotype, psychiatric symptoms, MnSOD, GPX, SOD2, CAT, polymorphism, 8-iso-PGF2α, 8-OHdG, total antioxidant capacity, glutathione

## Abstract

Wilson’s disease (WD) is an autosomal recessive disorder of copper metabolism caused by mutations in the *ATP7B* gene. While hepatic manifestations are frequent, psychiatric symptoms occur in up to 30% of patients and may precede neurological signs. This study was the first to assess the relationship between oxidative stress, selected genetic polymorphisms, and psychiatric symptoms in WD. A total of 464 patients under the care of the Institute of Psychiatry and Neurology in Warsaw were studied. Genotyping for *GPX1* (rs1050450), *SOD2* (rs4880), and *CAT* (rs1001179) was performed, along with biochemical analyses of copper metabolism, oxidative DNA, lipid and protein damage, and systemic antioxidant capacity. Among the most important observations are the following: the homozygous *GPX1* rs1050450 TT and *SOD2* rs4880 CC genotypes were associated with the lowest prevalence of psychiatric symptoms. The *CAT* rs1001179 TT genotype was linked to a delayed onset of psychiatric symptoms by 6.0–8.5 years. Patients with or without psychiatric symptoms did not differ significantly in saliva 8-OHdG, total antioxidant capacity, serum glutathione (GSH), catalase, and MnSOD; however, patients reporting psychiatric symptoms had significantly higher prostaglandin F2α 8-epimer (8-iso-PGF2α) concentrations and tended to have lower serum glutathione peroxidase (Gpx) concentrations compared to those without such symptoms. Our data firstly provide consistent evidence that oxidative stress balance associated with copper overload in the CNS may be associated with CNS damage and the development of psychiatric symptoms of WD. In particular, our findings of increased oxidative lipid damage together with decreased Gpx activity indirectly suggest that damage to neuronal membrane lipids, which may be potentially related to abnormalities in GSH metabolism, may have an etiological role in CNS damage and related symptoms.

## 1. Introduction

Wilson’s disease (WD; OMIM #277900) is an autosomal recessive disorder caused by pathogenic mutations in the *ATP7B* gene, impairing copper (Cu) metabolism. This defect leads to progressive Cu accumulation—initially in the liver, then in other organs including the brain, kidneys, cornea, and heart—manifesting in a diverse clinical picture, including hepatic, neurological, and psychiatric symptoms that may appear in isolation or in combination [1,2,3].

Hepatic presentation is the most common initial phenotype, observed in over half of patients. It ranges from asymptomatic transaminase elevations to chronic active hepatitis, cirrhosis with portal hypertension, or acute liver failure often accompanied by hemolytic anemia and renal injury. Typical findings include elevated aminotransferases, jaundice, ascites, splenomegaly, hypoalbuminemia, and encephalopathy. Neurological and psychiatric manifestations, present in approximately 30–50% of cases, include tremor, dystonia, ataxia, dysarthria, rigidity (often with Kayser–Fleischer rings), and psychiatric disturbances [4,5,6,7]. These encompass five broad categories—cognitive impairment, personality alterations, mood disorders, psychosis, and other psychiatric symptoms—with depression occurring in 20–60% of patients [8,9,10] and a significant suicide risk [8]. Other mood disorders, such as hypomania, frank mania, and bipolar disorder have also been reported [11,12,13,14,15]. Behavioral changes are seen in up to 71% of cases; psychosis or catatonia affect ~8% [16,17,18,19]. Cognitive impairment is typically mild (<25%) but can progress with disease chronicity [19,20,21,22].

Psychiatric symptoms alone initiate the disease in ~20% of patients and dominate in ~30%, frequently resulting in diagnostic delays of over two years. The neuropsychiatric phenotype of WD correlates with a fourfold increase in mortality, diminished quality of life, and poor adherence to chelation therapy [23,24,25,26,27].

Despite clear clinical impact, the pathogenesis of psychiatric features in WD remains poorly understood. In addition to Cu-induced neurotoxicity, altered dopaminergic signaling, and hepatic dysfunction, oxidative stress is increasingly implicated. Excess Cu catalyzes Fenton chemistry, generating reactive oxygen species (ROS), that damage lipids, proteins, and DNA. Oxidative harm can disrupt neuronal signaling and synaptic plasticity in regions critical for mood, cognition, and behavior [28,29,30,31,32,33,34,35]. As ROS-based mechanisms are central in other psychiatric disorders [36,37,38], we hypothesize that oxidative stress plays a major role in WD-related psychiatric morbidity. To test this, we analyzed oxidative stress parameters in WD patients with and without psychiatric symptoms. Specifically, we assessed genetic polymorphisms in antioxidant enzymes, quantified systemic markers of oxidative damage to lipids, proteins, and DNA, and measured the levels of both low-molecular-weight and large-molecular-weight enzymatic antioxidants and total antioxidant capacity (TAC).

## 2. Results

### 2.1. Genetic Studies

Psychiatric symptoms were observed in 157 (74 men (47.1%) and 87 women (52.9%)) of 464 individuals (33.8%) included in the genetic analysis of single nucleotide polymorphisms (SNPs) in genes encoding enzymatic antioxidants. The median age at the onset of psychiatric symptoms was 28.0 years (interquartile range [IQR], 14.0). In 74 patients (47.1% of those with psychiatric symptoms; 15.9% of the total cohort), psychiatric manifestations were present at the initial presentation of WD, including 34 men (45.9%) and 40 women (54.1%).

In 121 patients (77.1%), psychiatric symptoms co-occurred with various neurological manifestations, while isolated psychiatric symptoms were reported in 36 patients (22.9%). Cognitive impairment coexisting with other psychiatric symptoms was noted in 35 individuals. The most frequently diagnosed psychiatric disorders were mood disturbances, followed by cognitive impairment and personality disorders. Psychotic symptoms and other psychiatric conditions were observed only in a few individuals, as detailed in Table 1.

#### 2.1.1. Antioxidant Gene Polymorphisms and the Prevalence of Psychiatric Symptoms in WD Patients

Genotype frequencies for *CAT* rs1001179 SNP (c.-262C>T) and *SOD2* rs4880 (c.-9T>C [p.Val16Ala]) SNPs conformed to the Hardy–Weinberg equilibrium (HWE) in this cohort (Table 1). However, the *GPX1* rs1050450 SNP (c.593C>T [p.Pro198Leu]) genotype distribution deviated significantly from HWE (*p* < 0.04). Nearly equal numbers of individuals carried the p.198 Pro/Pro and Pro/Leu genotypes (216 (45.5%) and 223 (46.9%), respectively), while the Leu/Leu genotype was identified in only 36 patients (7.6%).

**Figure 1:**
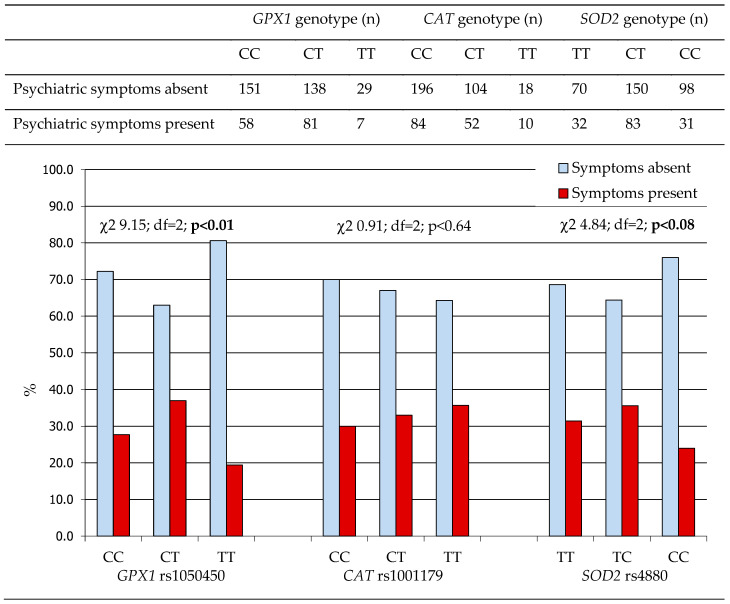
The prevalence of psychiatric symptoms among WD patients according to the *GPX1* rs1050450, *CAT* rs1001179, and *SOD2* rs4880 SNP genotype. *CAT*, gene encoding catalase; *GPX1*, gene encoding glutathione peroxidase; *SOD2*, gene encoding manganese superoxide dismutase.

No significant association was found between the *CAT* rs1001179 SNP and the overall prevalence of psychiatric symptoms in WD patients. Notably, the homozygous c.593TT (p.198 Leu/Leu) genotype of *GPX1* rs1050450 SNP and the homozygous c.-9 CC (p.16 Ala/Ala) genotype of *SOD2* rs4880 SNP were both associated with the lowest prevalence of psychiatric symptoms compared to other genotypes of the respective genes (Figure 1).

**Figure 2:**
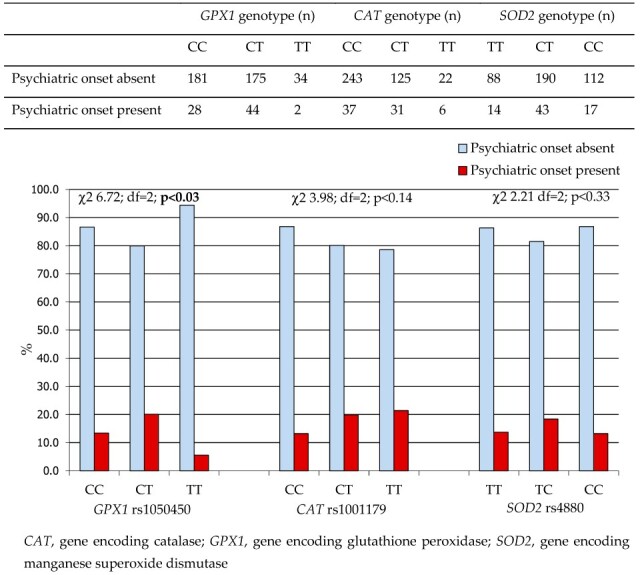
The prevalence of psychiatric onset in WD patients according to the *GPX1* rs1050450, *CAT* rs1001179, and *SOD2* rs4880 SNP genotype.

The *GPX1* rs1050450 c.593 TT (p.198 Leu/Leu) genotype was also associated with the lowest prevalence of psychiatric symptoms at the onset of WD (Figure 2). Among *CAT* genotypes, the c.-262 CC genotype appeared to be linked with the highest frequency of psychiatric symptoms at disease onset. In contrast, *SOD2* rs4880 genotypes did not demonstrate any significant association with the initial psychiatric presentation of WD.

Associations between individual genotypes and the prevalence of specific psychiatric disorders among WD patients are presented in Table 2a–c.

The *GPX1* rs1050450 c.593 TT (p.198 Leu/Leu) genotype tended to be associated with the lowest frequency of cognitive impairment, compared to the c.593 CC (p.198 Pro/Pro) and c.593 CT (p.198 Pro/Leu) genotypes; notably, none of the individuals with the TT genotype presented with psychotic symptoms. The *SOD2* rs4880 c.-9 TT (p.16 Val/Val) genotype appeared to have a protective effect against affective disorders. Additionally, an increasing dosage of the *CAT* rs1001179 c.-262 T allele was associated with a higher frequency of psychosis.

Due to the limited number of patients with individual psychiatric diagnoses, these findings should be interpreted with caution. The observed associations require validation in larger, independent cohorts to confirm their significance and clinical relevance.

#### 2.1.2. Antioxidant Gene Polymorphisms and Age at Onset of Psychiatric Symptoms in Patients with WD

No significant association was found between the *GPX1* rs1050450 or *SOD2* rs4880 genotypes and the age at the onset of psychiatric symptoms in WD patients. However, the c.-262 TT genotype of the *CAT* rs1001179 SNP was associated with a later onset of psychiatric symptoms, by approximately 6.0 and 8.5 years compared to the CC and CT genotypes, respectively, as presented in Table 3.

### 2.2. Biochemical Studies—Copper Metabolism, Oxidative Damage, and Antioxidant Parameters in WD Patients with and Without Psychiatric Symptoms

Of 33 WD patients in whom parameters related to Cu metabolism, oxidative damage to cellular structures, and natural antioxidant defense were analyzed, 20 were males (60.6%) and 13 were females (39.4%). Among them, 11 individuals (33.3%) exhibited psychiatric symptoms, with a median age at psychiatric onset of 36.0 years (interquartile range [IQR], 21.0). In all cases, psychiatric manifestations co-occurred with neurological symptoms. Cognitive impairment was reported in 7 patients, personality disorders in 3, affective disorders in 6, and other psychiatric symptoms in 1 patient. Notably, 3 patients presented with more than one psychiatric disorder. In 5 of the 11 symptomatic individuals (45.4%; 15.1% of the total analyzed subgroup), psychiatric symptoms were evident at the initial presentation of WD.

Patients with a history of psychiatric symptoms—either at disease onset or during the disease course—had significantly lower blood total Cu and ceruloplasmin (Cp) concentrations compared to those without psychiatric manifestations (Table 4).

Blood nitrotyrosine was detectable in 14 patients (10 without and 4 with psychiatric symptoms), with no significant difference observed between the two groups.

No significant differences were observed between patients with and without psychiatric symptoms in salivary 8-hydroxy-2’deoxyguanosine (8-OHdG), blood TAC, catalase (Cat), glutathione (GSH), or manganese superoxide dismutase (MnSOD) activity. However, patients reporting psychiatric symptoms showed significantly elevated levels of blood 8-epimer of prostaglandin F2α (8-iso-PGF2α), a marker of lipid peroxidation, and a trend toward lower blood glutathione peroxidase (GPx) concentrations, compared to those without psychiatric symptoms (Table 4).

## 3. Discussion

This study provides novel evidence linking oxidative stress to psychiatric manifestations in WD, demonstrating that variability in antioxidant defense—partly determined by genetic polymorphisms—may influence individual susceptibility. This research, by elucidating potential molecular mechanisms underlying psychiatric symptoms in WD, has implications not only for rare metabolic disorders but also for broader neuropsychiatric and personalized medicine fields.

We observed that psychiatric symptoms were least frequent in patients with the *GPX1* TT (Leu/Leu) genotype and most common in CT (Pro/Leu) heterozygotes. Although the T allele is associated with reduced GPx1 activity and greater vulnerability to oxidative stress [39,40,41], our results suggest a paradoxical protective role of the TT genotype in WD. This may reflect enhanced compensatory antioxidant responses, such as upregulation of Cat, peroxiredoxins, or mitochondrial adaptations [42,43], while heterozygotes may lack such robust compensation due to allele-specific effects. It is also plausible that moderate impairment in CT individuals fails to activate neuroprotective mechanisms, while more severe deficiency in TT homozygotes triggers adaptive responses; alternatively, diminished hydrogen peroxide detoxification in TT individuals may preserve redox signaling critical for neuronal plasticity and resilience [44,45]. These findings highlight the complexity of gene–environment interactions in WD and justify further functional studies.

The *SOD2* c.2734 CC (p.16Ala/Ala) genotype, associated with more efficient mitochondrial import and activity of MnSOD [46,47,48,49,50], was also linked to a lower prevalence of psychiatric symptoms. This result is consistent with reports of the protective role of the Ala/Ala genotype in schizophrenia [51], although other studies were inconclusive [52,53]. Additionally, *SOD2* variants have been implicated in methamphetamine-induced psychosis [54], likely due to overwhelmed MnSOD capacity in the presence of dopamine-derived ROS. Our findings align with the hypothesis that efficient mitochondrial ROS detoxification contributes to neuroprotection in WD.

The *CAT* c.-262C>T polymorphism was associated with age at onset of psychiatric symptoms, with TT carriers exhibiting a significantly delayed onset. The c.-262T allele increases *CAT* transcriptional activity [55], which may delay CNS damage under chronic oxidative stress. However, it was not associated with overall symptom presence, possibly due to limited *CAT* expression in vulnerable brain regions and its decline with neurodegeneration [56,57]. The protective effect may thus be peripheral, by reducing hepatic injury and delaying Cu release into circulation and the brain. Notably, in one of studies examining the role of *CAT* c.-262C>T in psychiatric disorders reported no significant association with age of onset, disease duration, or episode frequency in recurrent depressive disorder [58], suggesting a disease-specific or tissue-specific role for this polymorphism in WD.

Biochemically, patients with psychiatric symptoms showed higher levels of 8-iso-PGF2α and lower GPx activity. 8-iso-PGF2α, a reliable marker of lipid peroxidation [59], likely reflects oxidative injury to neuronal membranes, which are rich in polyunsaturated fatty acids. Such damage can impair neurotransmission, ion channel function, and membrane integrity, leading to neuronal dysfunction or death [60]. These results support the notion that lipid peroxidation contributes to CNS injury and symptom development. Prior studies in schizophrenia have reported similar correlations between lipid peroxidation and psychiatric symptom severity [61,62].

Interestingly, psychiatric symptoms in WD often emerge years after hepatic symptoms. This delay may be partly explained by the age-dependent decline in brain antioxidant capacity [63,64], increasing cerebral vulnerability to oxidative injury over time.

**Figure 3:**
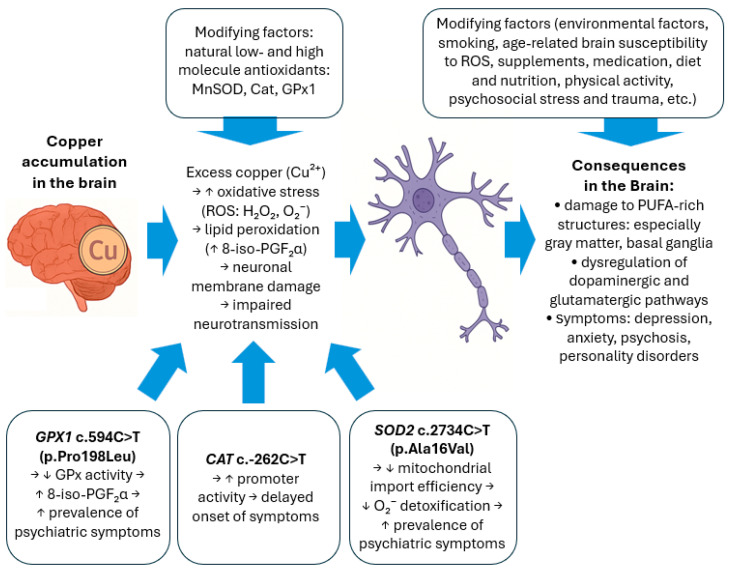
The mechanism of oxidative stress and its role in psychiatric symptoms in Wilson′s disease. *CAT*, gene encoding catalase; *GPX1*, gene encoding glutathione peroxidase; *SOD2*, gene encoding manganese superoxide dismutase; Cu, copper; 8-iso-PGF2α, prostaglandin F2α 8-epimer; PUFA, polyunsaturated fatty acids; ROS, reactive oxygen species, MnSOD, manganese superoxide dismutase; GPx, glutathione peroxidase; Cat, catalase.

A diagram illustrating the mechanistic model of the influence of oxidative stress and genetic factors on psychiatric symptoms in WD is presented in Figure 3.

Although psychiatric symptoms were more common in patients with lower serum Cu and ceruloplasmin, this likely reflects more severe Cu metabolism disturbances and increased oxidative stress, not a protective effect. Measuring non-ceruloplasmin-bound Cu (free Cu) would better clarify its role but was unavailable in our study.

Importantly, neurological and psychiatric symptoms in WD are distinct yet overlapping. While neurological symptoms (e.g., tremor, dystonia, dysarthria) result from basal ganglia and brainstem damage, psychiatric symptoms involve mood, cognition, and behavior, with shared anatomical and pathophysiological substrates. Oxidative stress may contribute to both symptom domains by damaging common brain structures. Understanding this overlap may support the development of integrated treatment strategies.

Our study has limitations. First, the sample size for enzymatic assays was limited. Second, we lacked data on non-ceruloplasmin Cu. Third, some patients had received antipsychotics prior to oxidative stress assessment, potentially affecting biomarker levels [65,66]. Finally, psychiatric syndromes are heterogeneous, requiring more rigorous and standardized assessments in future research, as recommended by Cai et al. [66].

Despite these limitations, our findings emphasize that oxidative stress, likely driven by Cu accumulation, may contribute to psychiatric symptoms in WD. In particular, impaired GSH metabolism and subsequent lipid peroxidation may underline CNS injury in WD. These insights highlight oxidative damage as a promising therapeutic target. Future studies should evaluate antioxidant-based interventions in WD. In the meantime, lifestyle strategies—including plant-based diets and smoking cessation—may provide ancillary benefits and merit inclusion in patient education. Such approaches could improve not only oxidative balance but also cardiometabolic health.

## 4. Materials and Methods

### 4.1. Patients

Our study included two distinct populations of patients with WD treated at the Institute of Psychiatry and Neurology in Warsaw, Poland.

The first—genetic—study group consisted of 464 patients with WD who underwent genetic testing for polymorphisms in genes encoding three major enzymatic antioxidants: MnSOD, Cat, and Gpx1. This cohort included both treated and untreated patients, as treatment status does not affect genotype. This population was analyzed to investigate whether specific genotypes are associated with the occurrence of psychiatric symptoms in WD.

The second—biochemical—study group comprised 33 newly diagnosed, treatment-naïve patients with WD, who had never received any Cu-depleting therapies. In this group, biochemical analyses were conducted to assess oxidative stress parameters, including markers of oxidative damage to DNA, lipids, and proteins; levels of low-molecular-weight antioxidants (e.g., GSH) and of major enzymatic antioxidants (MnSOD, Cat, and Gpx); and TAC. This cohort was used to examine whether oxidative stress markers differ between patients with a history of psychiatric symptoms (either at disease onset or at any time during the disease course) and those without psychiatric manifestations.

The diagnosis of WD was established based on the criteria proposed during the 8th International Meeting on Wilson’s Disease and Menkes Disease (so called Leipzig score) [67]. The Leipzig score includes evaluation of the following domains: clinical manifestations; presence of Kayser–Fleischer rings; Cu metabolism markers: serum Cp concentration and 24 h urinary Cu excretion; liver biopsy: Cu staining or quantitative hepatic Cu measurement; evidence of Coombs-negative hemolytic anemia; genetic testing for *ATP7B* mutations. A cumulative score ≥4 confirms the diagnosis of WD. This diagnostic algorithm is currently recommended by all major international hepatology societies, including the European Association for the Study of the Liver (EASL, 2025), the American Association for the Study of Liver Diseases (AASLD, 2023), and the European Society for Paediatric Gastroenterology, Hepatology and Nutrition (ESPGHAN, 2019) [68,69].

The onset of WD was determined using a standardized patient questionnaire and a comprehensive review of the available medical records. Data collection followed the protocol adopted for the EuroWilson project database (http://www.eurowilson.org), with minor modifications.

Psychiatric manifestations of WD were assessed by evaluating patients’ medical histories, self-reports of current or past psychiatric treatment, and review of available medical documentation to qualitatively confirm the presence of symptoms leading to a psychiatric diagnosis. In cases of diagnostic uncertainty, a psychiatric evaluation was conducted by one of the authors (T.S.), a board-certified psychiatrist, using the Mini-International Neuropsychiatric Interview (M.I.N.I.), a structured diagnostic tool for major psychiatric disorders (Polish version 5.0.0) [70,71]. Psychiatric disorders were diagnosed according to the international ICD-10 criteria (World Health Organization, 1993) [72], although no judgment was made regarding whether the psychiatric symptoms were primary or secondary to WD. We acknowledge that various psychometric tools are available to assess symptom severity; however, such quantitative analysis was beyond the scope of this study and not part of our research objectives. Our primary aim was to determine whether patients met diagnostic criteria for a given psychiatric disorder, not to measure the intensity of specific symptoms.

Psychiatric symptoms were categorized into four primary groups: (1) mood disorders/disturbances (including depression, mania, hypomania, and euphoria); (2) personality disorders; (3) cognitive impairment; (4) psychotic disorders. A fifth, heterogeneous category encompassed less frequent manifestations such as eating disorders, obsessive–compulsive disorder, attention-deficit/hyperactivity disorder, and anxiety disorders.

All patients provided written informed consent prior to participation in the study. The study protocol was approved by the Institutional Review Board of the Institute of Psychiatry and Neurology in Warsaw, Poland, and was conducted in accordance with the ethical principles outlined in the Declaration of Helsinki (1975).

### 4.2. Genetic Studies

#### Genotyping of Single Nucleotide Polymorphisms of the Glutathione Peroxidase 1 (*GPX1*), Catalase (*CAT*), and Manganese Superoxide Dismutase (*SOD2*) Genes

Peripheral blood samples were collected once from each participant. Genomic DNA was isolated from EDTA-treated blood samples either using TRI Reagent (Sigma-Aldrich, St. Louis, MO, USA; TRI Reagent is a registered trademark of Molecular Research Center, Inc.) or the Maxwell^®^ 16 System (Promega Corporation, Madison, WI, USA), depending on sample availability and processing logistics. All isolations were performed strictly according to the manufacturers’ protocols, ensuring reproducibility and quality of the extracted DNA. The DNA yield varied depending on the method used. TRI Reagent typically yielded 10–30 µg of DNA per 1 mL of EDTA-treated whole blood, while the Maxwell 16 system provided a more consistent yield in the range of 30–60 µg per 1 mL, with high purity suitable for molecular analyses.

The following SNPs were analyzed: (1) *GPX1* C>T substitution at nucleotide position 594 changing proline (Pro) to leucine (Leu) at codon 198 (Pro198Leu); SNP cluster reference number (NCBI SNP ID): rs1050450; (2) *CAT* C>T substitution at position −262 in the promoter region; NCBI SNP ID rs1001179; (3) *SOD2* C>T substitution at position 2734 changing alanine (Ala) to valine (Val) at residue 16 (Ala16Val); NCBI SNP ID: rs4880. Genotyping of *GPX1* rs1050450, *CAT* rs1001179, and *SOD2* rs4880 was performed using polymerase chain reaction/restriction fragment length polymorphism (PCR/RFLP) technology according to the methods described by Xiong et al. [73], Suzen et al. (2010) [74], and Lee et al. (2006) [75], respectively.

### 4.3. Biochemical Studies

#### 4.3.1. Analysis of Copper Metabolism

Serum Cp was measured by a colorimetric enzymatic assay as previously described [76]. Serum copper was determined by atomic adsorption spectroscopy.

#### 4.3.2. Analysis of Selected Peripheral Markers of Oxidative Damage to DNA, Lipids, and Proteins

Biochemical studies of peripheral markers of copper metabolism, oxidative damage to DNA, lipids and proteins were performed in 33 patients with newly diagnosed WD, who had never been treated with decoppering therapy. Saliva samples were collected using the Salivette^®^ system (Sarstedt, Nümbrecht, Germany), a standardized method for diagnostic and therapeutic saliva sampling, according to the manufacturer′s instructions. Peripheral blood was collected either into clot-activator tubes or into tubes containing the anticoagulant EDTA. Serum or plasma was separated according to the reagent manufacturers′ protocols (names of manufacturers and details of the reagents used are given in the following subsections of the methodology section) and stored at −70 °C until analysis.

The following parameters of oxidative damage to various cellular structures were analyzed: (i) plasma prostaglandin F2α 8-epimer (8-iso-PGF2α) as a strong marker of oxidative damage to cell membranes; the study was performed using the Direct 8-iso-Prostaglandin F2α ELISA kit (catalog number ADI-901-091; Enzo Life Sciences, Farmingdale, NY, USA); detection limit: 40 pg/mL; (ii) plasma 3-nitrotyrosine, as a marker of the oxidative modification of proteins; the tests were performed using the Enzyme Immunoassay for Nitrotyrosine kit (cat. no. 21055; OxisResearch, Portland, OR, USA); detection limit: 2 nM; (iii) saliva 8-hydroxy-2′deoxyguanosine (8-OHdG), a product of oxidative DNA damage; the tests were performed using the 8-hydroxy-2-deoxy Guanosine EIA Kit (cat. no. 589320; Cayman Chemical, Ann Arbor, MI, USA); detection limit: 33 pg/mL.

#### 4.3.3. Analysis of Systemic Antioxidant Capacity and Selected Small and Large Antioxidant Molecules

Biochemical tests of antioxidant capacity parameters were performed in 33 patients with newly diagnosed WD who had never been treated with decoppering drugs. The TAC of serum was assessed using the AOP-450 kit (cat. no. 21053; OxisResearch, Portland, OR, USA). The activity of manganese superoxide dismutase (MnSOD) in plasma was tested using the Superoxide Dismutase Assay Kit (cat. no. 706002; Cayman Chemical, Ann Arbor, MI, USA); test range: 0.025–0.25 units/mL SOD. Plasma glutathione peroxidase (GPx) activity was assayed using the Glutathione Peroxidase Assay Kit (cat. no. 703102; Cayman Chemical, Ann Arbor, MI, USA); assay range: 50–344 nmol/min/mL. Plasma Cat activity was assayed using the Catalase Assay Kit (cat. no. 707002; Cayman Chemical, Ann Arbor, MI, USA); assay range: 2–35 nmol/min/mL. Plasma glutathione concentration was assayed using the Glutathione Assay Kit (cat. no. 703002; Cayman Chemical, Ann Arbor, MI, USA); assay range: 0–16 μM GSH.

### 4.4. Statistical Analysis

To check whether the studied SNPs were in the Hardy—Weinberg equilibrium (HWE) in our study population, expected genotype numbers were calculated from allele frequencies, and the deviation from observed genotype numbers was determined using the χ2 test (we used the HWE calculator by Michael H. Court; https://view.officeapps.live.com/op/view.aspx?src=http%3A%2F%2Fwww.dr-petrek.eu%2Fdocuments%2FHWE.xls&wdOrigin=BROWSELINK; accessed on 15 April 2025). To check the difference in genotype frequencies between WD patients and other European populations, as reported in the National Center for Biotechnology Information database (http://www.ncbi.nlm.nih.gov), which was accessed on 4 March 2013, we used the “other tests for relevance” application in STATISTICA 14.0 (Cloud Software Group, Inc., Austin, TX, USA; 2023). The normality of the analyzed continuous variables was determined using the Kolmogorov–Smirnov and Lilliefors tests. Since individual variables were not normally distributed in most groups, means and interquartile ranges (IQR) were used for their characteristics, and the Kruskal–Wallis nonparametric analysis of variance (with post hoc test using the Mann–Whitney U test) or the Mann–Whitney U test (two-way variables) were used to compare groups. Categorical variables were compared between groups using the χ2 test with Yate′s correction, when appropriate, or the Fisher exact test. The following elements were analyzed in the phenotypic indices of WD symptoms: presence/absence of neuropsychiatric symptoms at the beginning of WD, as well as during the patients′ lifetime before the start of anti-copper treatment; age of patients at the onset of psychiatric symptoms of WD. A *p*-value of <0.05 was considered statistically significant.

## Figures and Tables

**Table 1:** Baseline patients’ characteristics and genotype distributions of *GPX1* rs1050450, *CAT* rs1001179, and *SOD2* rs4880.

Characteristics			
Sex, female/male, N (%)	254 (54.7)/210 (45.2)		
Age in years at onset of WD, median (IQR) (N = 420) ^a^	23.9 (13)		
The presence of psychiatric symptoms, n (%) (N = 464)	157 (33.8)		
The type of psychiatric symptoms ^b^ (N = 464)			
behavioral or personality disorders	39 (8.4)		
mild cognitive impairment	40 (8.6)		
psychotic disorder	7 (1.5)		
mood disorder	91 (19.6)		
others (apathy, sleep disturbances, irritability and emotional lability, impulsivity, anxiety disorders, obsessive– compulsive symptoms, attention deficits)	15 (3.2)		
Age in years at manifestation of psychiatric symptoms, median (IQR) (N = 125) ^a^	27.0 (17.0)		
The presence of psychiatric symptoms at WD onset, N (%) (N = 464)	74 (15.9)		
*GPX1* rs1050450 (c.593 C>T [p.Pro198Leu]) genotype, N (%) ^c^	CC (wt)	209 (45.0)	HWE χ^2^ 4.31; df 2; *p* < 0.04
	CT (het)	219 (47.2)	
	TT (var)	36 (7.8)	
*CAT* rs1001179 (c.-262C>T) genotype, N (%) ^c^	CC (wt)	280 (60.3)	HWE χ^2^ 0.99; df 2; *p* < 0.32
	CT (het)	156 (33.6)	
	TT (var)	28 (6.1)	
*SOD2* rs4880 (c.-9 T>C [p.Val16Ala]) genotype, N (%) ^c^	TT (wt)	102 (22.0)	HWE χ^2^ 0.02; df 2; *p* < 0.87
	TC (het)	233 (50.2)	
	CC (var)	129 (27.8)	

Ala, alanine; *CAT*, gene encoding catalase; C, cytosine; *GPX1*, gene encoding glutathione peroxidase; het, heterozygous wild-type/variant genotype; HWE, Hardy–Weinberg equilibrium; IQR, interquartile range; Leu, leucine; Pro, proline; *SOD2*, gene encoding manganese superoxide dismutase; T, thymine; Val, valine; var, homozygous variant genotype; wt, homozygous wild-type genotype; ^a^ for some patients, data on the age of onset of symptoms were not available; ^b^ the total number exceeds the number of subjects due to the presence of comorbidity; **^c^** the number of subjects and percentages are shown, as well as *p* value for Hardy–Weinberg equilibrium (HWE) testing; for alleles, the number of alleles and percentage are shown.

**Table 2:** The prevalence of psychiatric onset in WD patients according to the *GPX1* rs1050450 (**a**), *CAT* rs1001179 (**b**), and *SOD2* rs4880 (**c**) SNP genotype.

(**a**) *GPX1* rs1050450.					
Psychiatric Symptoms at WD Onset		*GPX1* rs1050450 genotype; n (%)			
		CC	CT	TT	statistics
Mood disorder	absent	173 (82.8)	171 (78.1)	29 (80.6)	χ^2^ 1.49; df 2 *p* < 0.47
	present	36 (17.2)	48 (21.9)	7 (19.4)	
Cognitive impairment	absent	194 (92.3)	195 (89.0)	35 (97.2)	χ^2^ 3.63; df 2 *p* < 0.16
	present	15 (7.2)	24 (11.0)	1 (2.8)	
Personality disorders	absent	193 (92.3)	197 (89.9)	35 (97.2)	χ^2^ 2.40; df 2 *p* < 0.30
	present	16 (7.7)	22 (10.8)	1 (2.8)	
Psychosis	absent	209 (100.0)	212 (96.8)	36 (100.0)	χ^2^ 7.95; df 2 *p* < 0.02
	present	0 (0.0)	7 (3.2)	0 (0.0)	
Others	absent	202 (96.0)	212 (96.8)	35 (97.2)	χ^2^ 0.33; df 2 *p* < 0.98
	present	7 (3.3)	7 (3.2)	1 (2.8)	
(**b**) *CAT* rs1001179.					
Psychiatric Symptoms at WD onset		*CAT* rs1001179 genotype; n (%)			
		CC	CT	TT	statistics
Mood disorder	absent	232 (82.9)	118 (75.6)	23 (82.1)	χ^2^ 3.37; df 2 *p* < 0.18
	present	48 (17.1)	38 (24.4)	5 (17.9)	
Cognitive impairment	absent	261 (93.2)	138 (88.5)	25 (89.3)	χ^2^ 3.04; df 2 *p* < 0.21
	present	19 (6.8)	18 (11.5)	3 (10.7)	
Personality disorders	absent	253 (90.4)	147 (94.2)	25 (89.3)	χ^2^ 2.16; df 2 *p* < 0.34
	present	27 (9.6)	9 (5.8)	3 (10.7)	
Psychosis	absent	278 (99.3)	153 (98.1)	26 (92.9)	χ^2^ 7.35; df 2 *p* < 0.02
	present	2 (0.7)	3 (1.9)	2 (7.1)	
Others	absent	269 (96.1)	152 (97.4)	28 (100.0)	χ^2^ 1.59; df 2 *p* < 0.45
	present	11 (3.9)	4 (2.6)	0 (0.0)	
(**c**) *SOD2* rs4880.					
Psychiatric Symptoms at WD onset		*SOD2* rs4880 genotype; n (%)			
		TT	CT	CC	statistics
Mood disorder	absent	82 (80.4)	180 (77.2)	111 (86.0)	χ^2^ 4.07; df 2 *p* < 0.13
	present	20 (19.6)	53 (22.7)	18 (13.9)	
Cognitive impairment	absent	94 (92.2)	209 (89.7)	121 (93.8)	χ^2^ 1.87; df 2 *p* < 0.39
	present	8 (7.8)	24 (10.3)	8 (6.2)	
Personality disorders	absent	93 (91.2)	217 (93.1)	115 (89.1)	χ^2^ 1.75; df 2 *p* < 0.41
	present	9 (8.8)	16 (6.9)	14 (10.5)	
Psychosis	absent	99 (97.1)	230 (98.7)	128 (99.2)	χ^2^ 1.95; df 2 *p* < 0.37
	present	3 (2.9)	3 (1.3)	1 (0.8)	
Others	absent	95 (93.1)	228 (97.8)	126 (97.7)	χ^2^ 5.51; df 2 *p* < 0.06
	present	7 (6.9)	5 (2.1)	3 (2.3)	

**Table 3:** Age at onset of psychiatric symptoms according to the *GPX1* rs1050450, *CAT* rs1001179, and *SOD2* rs4880 SNP genotypes.

Age at Manifestation of Psychiatric Symptoms, Years, Median (IQR)						
*GPX1* rs1050450 genotype			*p*	*GPX1* rs1050450 allele T status		*p*
CC	CT	TT		T (−)	T (+)	
27.0 (16.0) [n = 50]	27.0 (18.0) [n = 71]	25.0 (23.0) [n = 4]	ns	27.0 (16.0) [n = 50]	27.0 (19.0) [n = 75]	ns
*CAT* rs1001179 genotype				*CAT* rs1001179 allele C status		
CC	CT	TT		C (−)	C (+)	
27.5 (15.0) [n = 72]	25.0 (17.0) [n = 45]	33.5 (16.0) [n = 8]	CC vs. CT < 0.02 CT vs. TT < 0.06	33.5 (16.0) [n = 8]	27.0 (17.0) [n = 117]	<0.07
*SOD2* rs1050450 genotype				*SOD2* rs1050450 allele T status		
TT	CT	CC		T (−)	T (+)	
31.0 (16.0) [n = 29]	26.0 (17.5) [n = 68]	28.0 (16.5) [n = 28]	ns	28.0 (19.0) [n = 28]	27.0 (17.0) [n = 97]	ns

C, cytosine; *CAT*, gene encoding catalase; *GPX1*, gene encoding glutathione peroxidase; *SOD2*, gene encoding manganese superoxide dismutase; IQR, interquartile range; T, thymine.

**Table 4:** Parameters of copper metabolism and of antioxidant capacity among WD patients with or without psychiatric symptoms.

	Psychiatric Symptoms Before Decoppering Treatment			Psychiatric Symptoms at WD Onset		
	No	Yes	*p*	No	Yes	*p*
	N = 22	N = 11		N = 28	N = 5	
Copper metabolism parameters, ^ƒ^ median (IQR)						
Serum copper, µg/dL	69.5 (12.0)	58.0 (49.0)	<0.04	68.0 (17.5)	36.0 (49.0)	<0.021
Serum ceruloplasmin, mg/dL	17.2 (4.5)	2.5 (31.4)	<0.0006	16.6 (7.5)	2.1 (13.2)	<0.032
Oxidative damage parameters, median (IQR)						
8-iso-PGF2α, pg/mL, median (IQR)	1149.5 (521.0)	1830.0 (1435.0)	<0.001	1286.0 (845.0)	1696.0 (375.0)	ns
OHdG, pg/mL, median (IQR)	10,280.0 (12,323.0)	5751.0 (5695.5)	ns	9080.0 (10,480.0)	9490.0 (40,497.5)	ns
Antioxidant capacity parameters, median (IQR)						
Total antioxidant capacity, median (IQR)	647.0 (194.0)	654.0 (93.0)	ns	657.5 (209.0)	652.5 (147.5)	ns
Serum glutathione, µmol/L, median (IQR)	0.0 (0.7)	0.8 (1.3)	ns	0.0 (0.9)	0.4 (1.0)	ns
Catalase, nmol/min/mL, median (IQR)	100.0 (87.5)	135.0 (50.0)	ns	101.0 (80.0)	112.5 (69.0)	ns
Glutathione peroxidase, nmol/min/mL, median (IQR)	90.4 (24.8)	74.7 (40.8)	<0.056	88.9 (29.3)	59.3 (35.8)	<0.06
MnSOD, units/mL, median (IQR)	13.2 (5.0)	13.9 (3.0)	ns	13.3 (4.3)	13.9 (2.6)	ns

^ƒ^ normal ranges were as follows: 25.0–45.0 mg/dL for serum ceruloplasmin, 70.0–140.0 µg/dL for serum copper; IQR, interquartile range; MnSOD, manganese superoxide dismutase; 8-iso-PGF2α, 8-epimer of prostaglandin F2α; OHdG, 8-hydroxy-2’deoxyguanosine.

## Data Availability

The data presented in this study are available on request from the corresponding author. The data are not publicly available due to ethical restrictions related to patient confidentiality.

## Abbreviations

The following abbreviations are used in this manuscript:

8-iso-PGF2α	Prostaglandin F2α 8-epimer
8-OHdG	8-hydroxy-2′deoxyguanosine
ATP7B	ATP-ase 7B
Cat	Catalase
CNS	Central Nervous System
Cu	Copper
DNA	Deoxyribonucleic Acid
GPx	Glutathione Peroxidase
GSH	Glutathione
HWE	Hardy–Weinberg equilibrium
IQR	Interquartile Range
MnSOD	Manganese Superoxide Dismutase
OMIM	Online Mendelian Inheritance in Man
ROS	Reactive Oxygen Species
SNP	Single Nucleotide Polymorphism
